# Impact of the revised definition on incidence and outcomes of acute exacerbation of idiopathic pulmonary fibrosis

**DOI:** 10.1038/s41598-022-12693-5

**Published:** 2022-05-25

**Authors:** Jung-Wan Yoo, Jehun Kim, Jin Woo Song

**Affiliations:** 1 Department of Internal Medicine, Gyeongsang National University Hospital, Jinju, Republic of Korea; 2 Department of Pulmonary and Critical Care Medicine, Asan Medical Center, University of Ulsan College of Medicine, 88, Olympic-Ro 43-Gil, Songpa-Gu, Seoul, 05505 Republic of Korea

**Keywords:** Outcomes research, Disease-free survival

## Abstract

The revised definition of acute exacerbation (AE) in idiopathic pulmonary fibrosis (IPF) was proposed in 2016, but changes in the incidence and impact on prognosis of the re-defined AE compared to those of the previous definition remain unclear. Clinical data of 445 patients with IPF (biopsy proven cases: 165) were retrospectively reviewed. The median follow-up period was 36.8 months and 17.5% (n = 78) experienced AE more than once. The 1- and 3-year incidence rates of AE were 6.7% and 16.6%, respectively, and idiopathic AE accounted for 82.1% of AE. Older age, lower diffusing capacity of the lung for carbon monoxide and 10% relative decline in forced vital capacity for 6 months were independently associated with AE. The in-hospital mortality rate following AE was 29.5%. In the multivariable analysis, AE was independently associated with poor prognosis in patients with IPF. Compared to the old definition, the revised definition relatively increased the incidence of AE by 20.4% and decreased the in-hospital mortality by 10.1%. Our results suggest that the revised definition affects approximately 20% increase in the incidences and 10% reduction in the in-hospital mortality of AE defined by the past definition.

## Introduction

Idiopathic pulmonary fibrosis (IPF) is a chronic progressive fibrosing interstitial lung disease of unknown etiology, with median survival of 3 years^1^. The natural course of IPF is variable and unpredictable; some patients have a relatively steady decline in lung function, punctuated by acute respiratory worsening, named acute exacerbation (AE)^2–4^. The incidence of AE ranged from 9.6 to 61% and AE was associated with a high mortality rate varying between 12 to 100%^5–10^.

In 2016, the revised diagnostic criteria of AE-IPF was proposed, which included any acute respiratory events characterized by acute or subacute worsening or development of dyspnea, typically within 1 month along with bilateral infiltration except for cardiogenic or volume overload pulmonary edema^11^. Based on the revised definition, a recent study reported that AE of IPF accounted for 30% of acute respiratory deterioration that required hospitalization (n = 106) and was significantly associated with 90-day mortality (hazard ratio [HR] 3.832, 95% confidence interval [CI] 1.528–9.611, *P* = 0.004)^12^. Most reports of AE have been investigated based on the old definition. However, compared to the old definition, the effect of the new definition on the incidence and outcome of AE has not been reported. As this is important for designing future clinical trials, our study aimed to investigate the incidence, risk factors, and outcomes of AE-IPF by the revised definition and compare them with those of the old definition.

## Methods

### Study population

Between January 2009 to December 2013, 514 patients with IPF who were diagnosed at Asan Medical Center, Seoul, Republic of Korea, were enrolled. All patients met the diagnostic criteria of the American Thoracic Society (ATS)/European Respiratory Society (ERS)/Japanese Respiratory Society/Latin American Thoracic Association consensus statement^13^. Among these, 69 patients who first presented with respiratory deterioration (RD) (n = 57), those treated for lung cancer (n = 11) or underwent lung transplantation (n = 1) were excluded. The remaining 445 patients with IPF (biopsy proven cases: 165) were finally included in this study (Fig. S1). The study protocol was approved by the Institutional Review Board of Asan Medical Center (approval number 2017-0915) and written informed consent was waived due to the retrospective nature of the study. All methods were performed in accordance with the relevant guidelines and regulations of the journal.

### Data collection

Clinical and survival data were retrospectively collected from electronic medical records, telephone interviews, and/or records of National Health Insurance of Korea. Forced vital capacity (FVC), diffusing capacity of the lung for carbon monoxide (DLco) and total lung capacity (TLC) were measured according to the ATS/ERS recommendation^14–16^, and the results were expressed as percentages of the normal predicted values. The 6-min walk test (6MWT) was performed according to the ERS/ATS guidelines^17^. Bronchoalveolar lavage (BAL) was performed based on ATS guideline^18^.

Acute respiratory deterioration (RD) was defined as an acute worsening of dyspnea requiring hospitalization with newly developed radiologic abnormalities^11,19^. AE was defined based on the revised criteria put forth by Collard et al. in 2016^11^ and further categorized as idiopathic or triggered AE, depending on whether underlying triggers could be identified. Patients who did not experience RD during follow-up were classified as the no-RD group, and those who experienced RD other than AE were categorized as the no-AE RD group. The suspected AE was defined as acute respiratory worsening in which all AE criteria could not meet due to missing data.

For microbiologic evaluation, we performed multiple microbiologic tests. Although BAL or endotracheal aspiration could not be performed in all patients with RD due to instability, at least sputum samples as one of respiratory samples were collected and tested in all patients. In addition to respiratory samples, blood and urine samples were tested for microbioloigic evaluation. Microbiological evaluation include viral antigen test using FITC-conjugated anti-virus polyclonal antibody for virus (respiratory syncytial virus, influenza virus, parainfluenza virus, adenovirus, human Metapneumovirus) or respiratory virus multiplex RT-PCR for virus (adenovirus, coronavirus 228E/NL63, OC43, parainfluenza virus 1, 2, 3, 4, rhinovirus A/B/C, Respiratory syncytial virus A, B, influenzavirus A, B, bocavirus 1/2/3/4, metapneumovirus, and enterovirus) and direct fluorescence monoclonal antibody staining for *Pneumocystis jiroveci* in BAL fluid; serologic tests for cytomegalovirus and species of *Mycoplasma*, *Legionella* and *Aspergillus*; and urinary antigen tests for *Streptococcus* *pneumoniae* and *Legionella*.

The past definition of AE was based on the diagnostic criteria proposed in 2007^3^. The relative changes in forced vital capacity (FVC) ≥ 10% from baseline for 6 months was defined as the disease progression (DP). The relative changes in FVC for 6 months from baseline were calculated as follows; (FVC % predicted _after 6 months _− FVC % predicted _baseline_)/FVC % predicted _baseline_ × 100%^20^.

### Statistical analysis

All values were expressed as mean ± standard deviation for continuous variables or percentages for categorical variables. The Student’s *t* test or the Mann–Whitney *U* test was used for continuous variables, and the Chi-squared test or Fisher’s exact test was used to compare categorical variables. We analyzed risk factors and mortality rates based on the first AE. Cox proportional hazards analysis was employed to identify predicting variables for AE or the overall mortality. Logistic regression analysis was undertaken to determine predicting factors of in-hospital mortality in patients with AE. Variables with *P* value < 0.1 in the unadjusted analysis were entered into the multivariable models with backward, stepwise elimination method. Survival analysis was conducted using the Kaplan–Meier method, and differences were assessed by the long-rank test. All *P* values were two-tailed, and those < 0.05 were considered statistically significant. Data analysis was performed using the Statistical Package for the Social Sciences software version 25.0 (SPSS Inc., Chicago, IL, USA).

## Results

### Incidence

**Figure 1:**
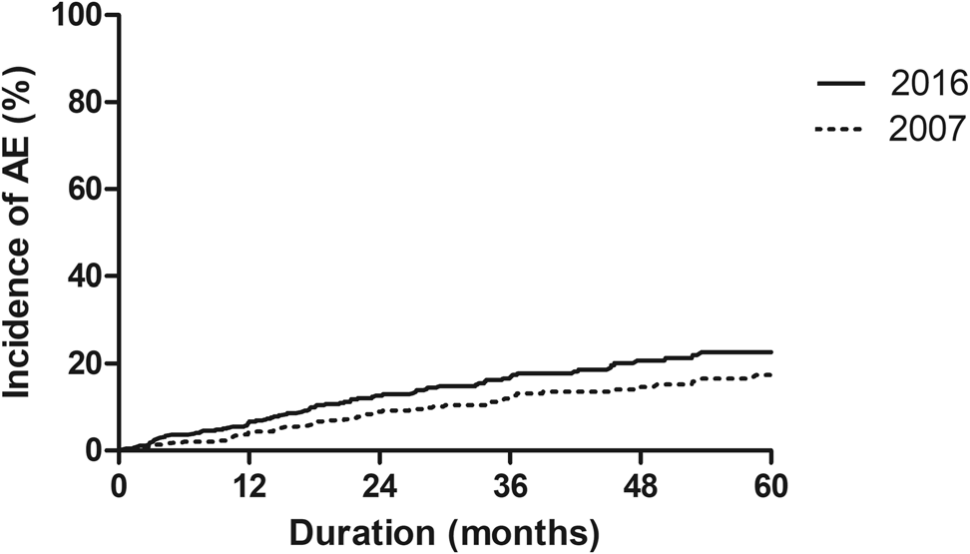
Comparison of incidence of AE of IPF according to 2007 and 2016 definition of AE. *AE* acute exacerbation, *IPF* idiopathic pulmonary fibrosis.

The patients’ mean age was 66.4 years, and 76.5% were male. The median follow-up period was 36.8 months (interquartile range 21.8–57.6 months). During follow-up, 119 (26.7%) patients experienced RD more than once (range 1–4 episodes), and 78 (65.5% of those with RD) experienced definite AE (range 1–3 episodes) (Table S1 and Fig. S2). The 1-, 2-, and 3-year cumulative incidence of the first AE were 6.7%, 12.6%, and 16.6%, respectively (Fig. 1) and incidence rate was 57.4/1000 person-years during follow-up period. The proportion of idiopathic and triggered AE was 82.1% (n = 64) and 17.9% (n = 14), respectively. Among 14 triggered AE, 12 were triggered by infection, followed by drug toxicity and post-operative condition (n = 1 each) (Table S1). AE tended to occur more frequently in winter and spring (n = 44, 56.4%) compared to the other seasons (n = 34, 43.6%) (Fig. S3).

### Risk factors

Patients in the RD group had higher CRP levels, lower lung function (FVC, DLco, and TLC), shorter distance and lower oxygen saturation (SpO_2_; resting and the lowest) during the 6MWT and more frequent DP than the no-RD group (Table 1). In the AE group, patients were of older age, had lower lung function, shorter distance, and lower SpO_2_ (resting and the lowest) during the 6MWT, and more frequent DP than those in the no-RD group.

**Table 1:** Comparison of the baseline characteristics between IPF patients with acute respiratory deterioration and those without.

Characteristics	RD			No-RD
	Total	AE	no-AE	
No. of patients	119	78	41	326
Age, years	67 ± 7.8	68.6 ± 7.9*	63.9 ± 6.9	66.1 ± 7.7
Male gender	84 (74.8)	56 (71.8)	33 (80.5)	251 (77)
Ever-smokers	80 (67.2)	51 (65.4)	29 (70.7)	241 (73.9)
BMI, kg/m^2^	24.1 ± 3.3	24.2 ± 3.2	23.9 ± 3.6	24.3 ± 3.1
Charlson comorbidity index	2.6 ± 1.2	2.9 ± 1.2	2.2 ± 1.1	2.7 ± 1.3
Serum CRP, mg/dl	1.2 ± 3.4* (n = 117)	0.9 ± 1.9* (n = 76)	1.8 ± 5.1 (n = 41)	0.5 ± 1.3 (n = 319)
**Lung function test**				
FVC, % pred	62.8 ± 15.6*	63.3 ± 15.6*	62.1 ± 15.8*	71.1 ± 15.5
DLco, % pred	49.3 ± 15.5*	49.3 ± 15.6*	49.4 ± 15.5*	57 ± 16.5
TLC, % pred	64.4 ± 13.2*	64.1 ± 13.6*	65.1 ± 12.8*	71.4 ± 12.9
**6MWT**	**N = 117**	**N = 76**	**N = 41**	**N = 326**
Distance, meter	384 ± 123*	369.1 ± 128.1*	411.7 ± 109	419.9 ± 106.2
Resting SpO_2_, %	95.6 ± 1.8*	95.7 ± 1.7*	95.5 ± 1.9*	96.4 ± 1.5
Lowest SpO_2_, %	88 ± 5.8*	87.5 ± 5.9*	88.9 ± 5.7*	90.8 ± 6.9
Disease progression^a^	30/107 (28)*	22/69 (31.9)*	8/38 (21.1)	38/275 (13.8)
Steroid ± IM ^b^	19 (16)*	15 (19.2)	4 (9.8)	84 (25.8)

Data are expressed as a mean ± standard deviation or a number (%) unless otherwise indicated.

*IPF* idiopathic pulmonary fibrosis, *RD* acute respiratory deterioration, *AE* acute exacerbation, *BMI* body mass index, *CRP* C-reactive protein, *FVC* forced vital capacity, *DLco* diffuse lung capacity of carbon monoxide, *TLC* total lung capacity, *6MWT* 6-min walk test, *SpO*_*2*_ saturation of pulse oximetry, *IM* immunosuppressant.

*P < 0.05 compared to no-RD.

^a^Disease progression was defined as 10% relative decline in FVC for 6 months.

IM^b^: Azathioprine (n = 26), Mycophenolate mofetil (n = 12), Cyclosporine (n = 10).

In the unadjusted Cox regression analysis, older age, lower lung function, shorter distance, and lower SpO_2_ (resting and the lowest) during the 6MWT, and DP were significantly associated with occurrence of AE in patients with IPF (Table 2). In the multivariable analysis, older age, lower DLco, and DP were independently associated with AE-IPF.

**Table 2:** Risk factors for AE in patients with IPF assessed by using Cox regression analysis.

Variables	Unadjusted			Multivariable		
	HR	95% CI	*P* value	HR	95% CI	*P* value
Age	1.054	1.022–1.087	0.001	1.046	1.012–1.081	0.008
Male gender	0.761	0.465–1.246	0.278			
BMI	0.973	0.902–1.049	0.470			
Ever smoker	1.434	0.899–2.287	0.130			
FVC, % pred	0.960	0.945–0.975	< 0.001	–	–	–
DLco, % pred	0.963	0.950–0.977	< 0.001	0.968	0.953–0.984	< 0.001
6MWT distance	0.995	0.993–0.997	< 0.001	–	–	–
6MWT resting SpO_2_	0.799	0.701–0.911	0.001	–	–	–
6MWT lowest SpO_2_	0.971	0.957–0.984	< 0.001	–	–	–
Disease progression^a^	3.401	2.038–5.676	< 0.001	3.293	1.946–5.571	< 0.001
Steroid ± IM	0.792	0.451–1.392	0.418	–	–	–

TLC was excluded for analysis due to close correlation with FVC (r = 0.890).

*HR* hazard ratio, *CI* confidence interval, *AE* acute exacerbation, *IPF* idiopathic pulmonary fibrosis, *BMI* body mass index, *FVC* forced vital capacity, *DLco* diffuse lung capacity of carbon monoxide, *TLC* total lung capacity, *6MWT* 6-min walk test, *SpO*_*2*_ saturation of pulse oximetry, *IM* immunosuppressant.

^a^Disease progression was defined as 10% relative decline in FVC for 6 months.

### Survival during hospitalization

During hospitalization, patients with AE (n = 78) showed a higher in-hospital mortality rate (29.5% vs*.* 9.8%, *p* = 0.015) than those with no-AE RD (n = 41). The in-hospital mortality between idiopathic and triggered AE groups did not differ (32.8% vs*.* 14.3%, *p* = 0.211). Among the patients with AE, the non-survivors, at the time of hospitalization, had shorter duration of dyspnea before hospitalization, more frequent fever, higher CRP levels, and lower arterial oxygen partial pressure/fractional inspired oxygen (P/F) ratio than the survivors (Table S2). For treatment of AE, all 78 patients received empirical antibiotics, 57 (73.1%) received intravenous corticosteroid therapy (9 with steroid pulse [500 mg/d or more of methylprednisolone], 38 with high dose steroid [0.5 mg/kg/d or more of methylprednisolone]) and 10 with low dose steroid [less than 0.5 mg/kg/d of methylprednisolone]) for treatment of AE. In addition, 16 patients (20.5%) were treated with immunosuppressive agents (10 with cyclosporine, 5 with azathioprine and 1 with mycophenolate mofetil). There was no significant differences of treatment between the survivors and non-survivors during hospitalization in patients with AE (Table S3).

In the unadjusted logistic analysis, shorter duration of dyspnea, fever, higher CRP levels, and lower P/F ratio, and more frequent use of steroid before AE were significantly associated with the in-hospital mortality in IPF patients with AE (Table S4). In the multivariable analysis, lower P/F ratio was the only independent prognostic factor toward in-hospital mortality in IPF patients with AE. Survival rates after hospitalization between idiopathic and triggered cases (median survival period, 4.1 months vs. 5.5 months, *p* = 0.656; Fig. S4) did not differ.

### Impact on overall survival

**Figure 2:**
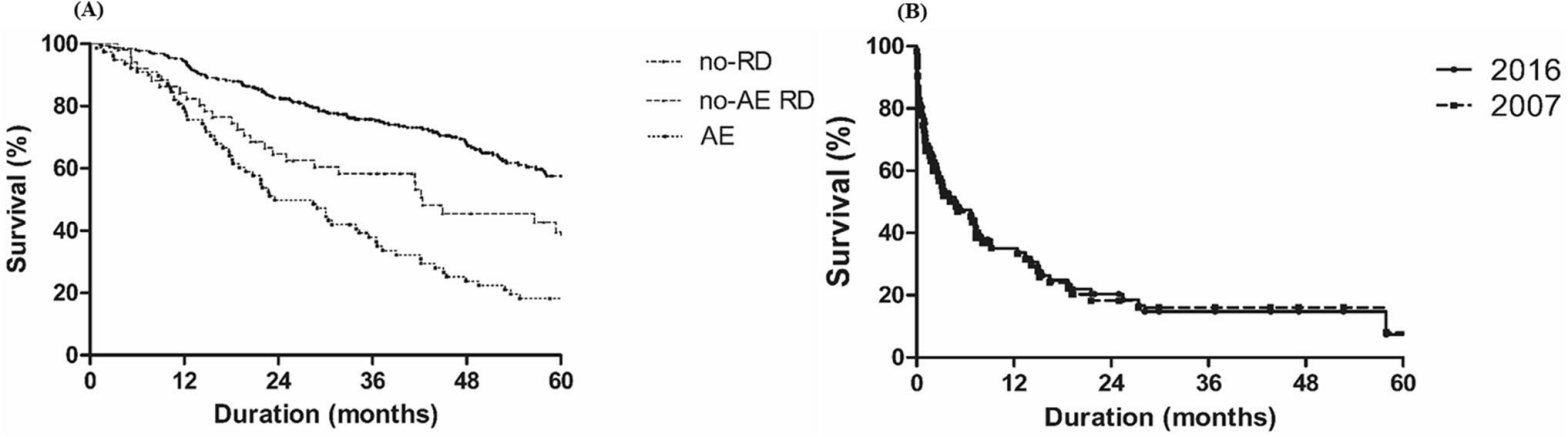
(**A**) Comparison of survival curves from diagnosis of IPF between AE, no-AE RD and no-RD groups among patients with IPF. (**B**) Comparison of survival curves after hospitalization in patients with AE according to 2007 and 2016 definition of AE. *AE* acute exacerbation, *RD* respiratory deterioration, *IPF* idiopathic pulmonary fibrosis.

During follow-up, 207 (46.5% of total subjects) patients with IPF died. There were significant differences in overall survival from the diagnosis of IPF between patients experienced AE (median survival period: 23.5 months) and others (vs. 42.4 months [no-AE RD], *p* = 0.017; vs. not reached [no-RD], *p* < 0.001; Fig. 2A).

In the unadjusted Cox regression analysis, older age, lower body mass index (BMI), Charlson comorbidity index, FVC and DLco, shorter distance, and lower SpO_2_ (resting and the lowest) during the 6MWT, DP, and AE were significantly associated with poor prognosis in patients with IPF (Table 3). Following multivariable analysis, AE was independently associated with poor prognosis (HR, 1.740; 95% CI, 1.220–2.481, *p* = 0.002), along with older age, lower BMI and FVC, DP, shorter distance and lower the minimum SpO_2_ during 6MWT.

**Table 3:** Prognostic factors for overall mortality in patients with IPF assessed by using Cox regression analysis.

Variables	Unadjusted			Multivariable		
	HR	95% CI	*P* value	HR	95% CI	*P* value
Age	1.040	1.021–1.060	< 0.001	1.028	1.005–1.052	0.018
Male gender	0.984	0.713–1.360	0.924			
BMI	0.908	0.865–0.953	< 0.001	0.944	0.894–0.997	0.037
Charlson comorbidity index	1.130	1.018–1.255	0.022	–	–	–
Ever smoker	0.957	0.706–1.297	0.777			
FVC, % pred	0.946	0.937–0.956	< 0.001	0.974	0.961–0.987	< 0.001
DLco, % pred	0.951	0.943–0.960	< 0.001	0.987	0.973–1.001	0.066
6MWT distance	0.994	0.993–0.996	< 0.001	0.998	0.997–1.000	0.038
6MWT resting SpO_2_	0.791	0.728–0.858	< 0.001	–	–	–
6MWT lowest SpO_2_	0.968	0.960–0.976	< 0.001	0.973	0.957–0.990	0.002
Disease progression^a^	3.043	2.169–4.267	< 0.001	2.269	1.568–3.283	< 0.001
AE^b^	3.250	2.387–4.425	< 0.001	1.740	1.220–2.481	0.002
Steroid ± IM	1.057	0.767–1.457	0.734			

*IPF* idiopathic pulmonary fibrosis, *HR* hazard ratio, *CI* confidence interval, *BMI* body mass index, *FVC* forced vital capacity, *DLco* diffuse lung capacity of carbon monoxide, *6MWT* 6-min walk test, *SpO*_*2*_ saturation of pulse oximetry, *AE* acute exacerbation, *IM* immunosuppressant.

^a^Disease progression was defined as 10% relative decline in FVC for 6 months.

^b^AE was compared to no respiratory deterioration in Cox regression analysis.

### Comparison of incidences, and outcomes

When data of all patients were re-analyzed based on the past definition of AE, it was noted that AE occurred in 64 (14.4%; 14 definite and 50 suspected) patients with IPF, and the 1-, 2- and 3-year cumulative incidences of AE were 4.2%, 8.9%, and 12%, respectively. The in-hospital mortality rates of patients with AE were 32.8%. While a lower P/F ratio was seen as an independent prognostic factor for in-hospital mortality in patients with AE (Table S5), AE was an independent prognostic factor for overall mortality in patients with IPF (HR, 2.978; 95% CI, 1.433–6.109, *p* = 0.003) (Table S6).

**Figure 3:**
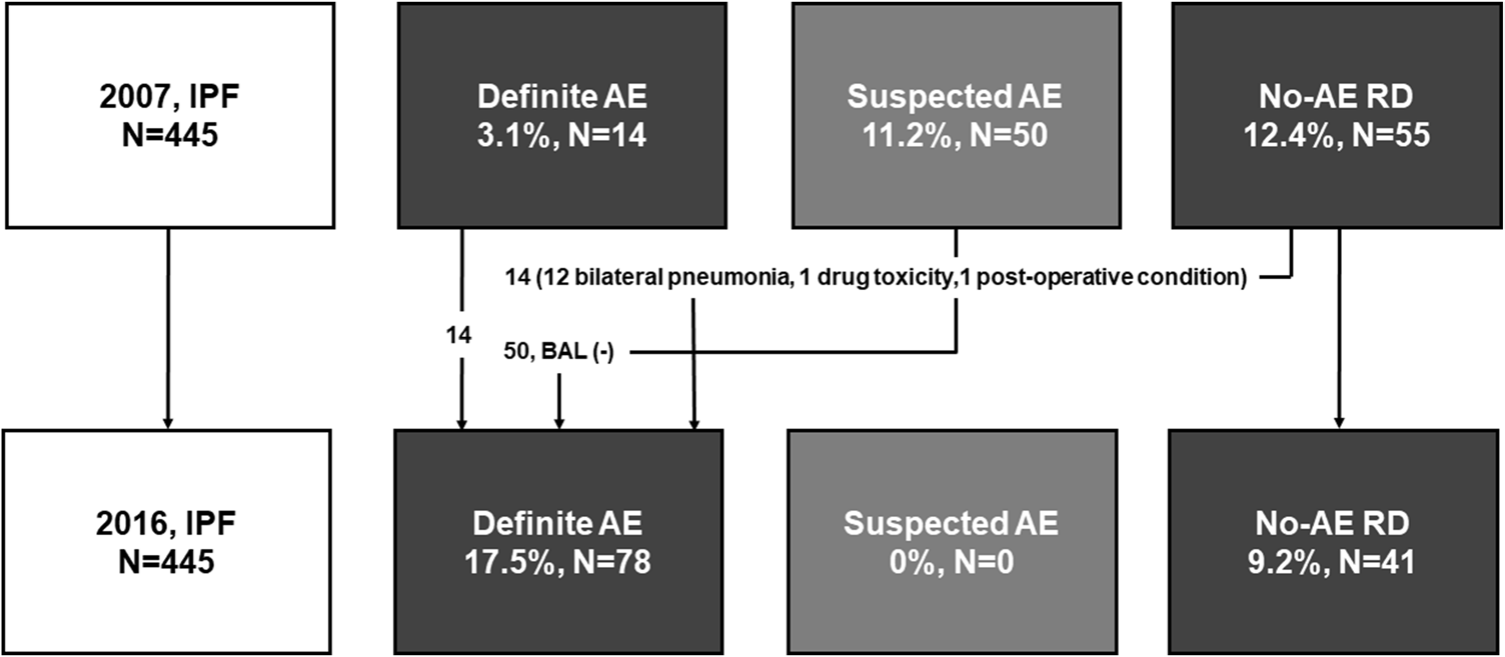
Change in the incidence of AE in patients with IPF according to 2007 and 2016 definition of AE. *IPF* idiopathic pulmonary fibrosis, *AE* acute exacerbation, *RD* respiratory deterioration.

The revised definition numerically increased the overall incidence of AE from 14.4 to 17.5% (relative increase of 20.4%) in patients with IPF (Fig. 3); 14 patients, not classified as AE but as no-AE RD by the past definition, were re-classified as triggered AE by the revised diagnostic criteria. There was no suspected case of AE as per the revised definition. The revised definition also decreased in-hospital mortality after AE from 32.8 to 29.5% (relative decrease of 10.1%) in patients with IPF; the 14 patients classified as triggered AE by the revised criteria, showed numerically lower in-hospital mortality rate (14.3% vs. 32.8%, *p* = 0.211) compared with those with idiopathic AE. However, overall mortality after hospitalization was similar between patients who experienced AE by both the definitions (81.3% vs. 82.1%, Fig. 2B).

## Discussion

In this study using the revised definition of AE, the 1-, 2-, and 3-year incidences of AE were 6.7%, 12.6%, and 16.6%, respectively. Older age, lower DLco and DP (10% relative decline in FVC for 6 months) were predictors for AE. AE was associated with a poor prognosis and appeared to have a significant impact on overall survival in patients with IPF. The revised criteria increased incidence of AE by 20.4% compared to those by the previous definition, and decreased the in-hospital mortality by 10.1%.

Several studies^12,21,22^ have reported incidence of AE based on the revised definition of AE. A study that included 225 patients with IPF, reported that the 1-year incidence of AE was 7.6%^12^. Okuda et al., in 107 patients with biopsy-proven IPF, also documented that cumulative incidence of AE was 9.6% for 1 year, 16.8% for 2 year and 23.9% for 3 years^21^. Moreover, Suzuki et al., using fibrotic ILD cohort (n = 1019), showed that AE occurred in 24% of patients with IPF (n = 462) during follow-up (median period: 3.4 years)^22^. Though these results were in line with our findings, they did not compare the results obtained using the new criteria with those of the older one.

In our study, older age, lower DLco and DP were independent predictors for AE-IPF. As documented in previous reports, lower lung function is the well-known risk factor for AE^21–23^. The results of our study are consistent with this finding. However, in our cohort, older age was one of risk factors for AE-IPF, which was not shown in previous studies^21,22^. The revised definition includes AE triggered by infection. The elderly are more susceptible to respiratory infections due to age-related dysfunction of the immune system (decrease in innate and adaptive immunity), and blunting of cough reflex or decline in mucociliary clearance^24^. These suggest that older age can be considered as a plausible risk factor of AE in our study. These results suggest that early diagnosis of IPF and early use of antifibrotic agents, and influenza or pneumococcal vaccination might be a useful prevention strategy of AE in patients with IPF^25^. Blood biomarkers (CRP, LDH, and total cholesterol) were also suggested to be useful in predicting prognosis in patients with AE-IPF^26^.

The results of our study indicate that AE was an independent prognostic factor for overall mortality in patients with IPF. Some studies using the revised definition of AE also demonstrated that AE was the independent prognostic factors in patients with IPF^21,22^. Okuda et al., showed that 90-day survival rate of the AE group (n = 39) was 50.4%, significantly lower than that of the non-AE group (*P* < 0.001)^21^. Suzuki et al., also, reported that AE was an independent predictor for mortality (HR 1.868, 95% CI 1.378–2.533, *P* < 0.001)^22^.

In our study, there was no difference of in-hospital mortality between idiopathic and triggered AE, which supports the validity of the revised definition of AE. Except for one study conducted by Kishaba et al.^27^, several studies, using the revised definition of AE, also reported findings similar to our results^12,21,28^. Teramachi et al., in 35 IPF patients with AE, reported that there was no significant difference in 90-day mortality with respect to survival (42% vs. 55%; *P* = 0.478) between idiopathic (n = 24) and triggered AE (n = 11)^12^. Okuda et al., in 39 IPF patients with AE, also showed that there were no significant differences in 90-day survival rates (45% vs. 60%; *P* > 0.05) between idiopathic (n = 29) and triggered AE (n = 10)^21^. Moreover, Yamazoe et al., in 64 patients with AE-IPF, reported that there was no difference in terms of in-hospital mortality (52.4% vs*.* 59.1%; P = 0.68) between idiopathic (n = 42) and triggered AE (n = 22)^28^. However, Kato et al. reported that, in their 79 patients with AE-idiopathic interstitial pneumonia (59 IPF and 20 unclassifiable interstitial pneumonia), patients with infection-triggered exacerbations (190 days, 95% CI = 10.157–369.853) had significantly longer median survival duration than that in those with idiopathic or non-infection-triggered AEIIPs (29 days, 95% CI = 12.057–43.493, *P* = 0.012)^29^. 90-day mortality rate was significantly lower in patients with infection-triggered AE than in patients with idiopathic or non-infection-triggered AE IIPs (44% vs 70.5% vs 75%, *P* = 0.022). A possible cause of reduced in-hospital morality of triggered AE might be that triggered events are relatively easier to correct than idiopathic cases.

In contrast to the 2007 diagnostic criteria on AE-IPF^3^, the revised definition emphasizes the pathophysiology rather than the cause of acute deterioration; any acute deterioration showing the pathophysiology of acute lung injury was defined as an AE. Therefore, the revised definition do not require invasive diagnostic procedures to diagnose AE, and has changed our clinical practice in terms of avoiding invasive procedure to diagnose AE in patients with AE-IPF^19,23^. This change seems to be practical for both clinical practice as well as clinical trials. In our study, the revised criteria relatively increased incidence of AE by 20.4% compared to that by the previous one. In addition, mortality rate decreased by 10%, which reflects real clinical situation in terms of clinical outcomes in IPF patients with AE. Our cohort also showed that all suspected AE by the past definition was included as definite AE in the revised definition. This finding suggests that more patients with IPF may be eligible for future clinical trials involving AE-IPF.

This study has some limitations. First, it was a retrospective observational study performed at a single center, which may have limited the generalizability of the results. However, baseline characteristics of our patients were similar to those included in previous reports^12,21,28^. Second, BAL or endotracheal aspiration was not performed in all patients with RD due to instability, but microbial tests, including respiratory virus PCR panels, were performed for all patients. Third, patients were diagnosed before the era of antifibrotic therapy, when anti-inflammatory agent such as corticosteroid was a standardized therapy. Because treatment with anti-fibrotic agents reduces AE in patients with IPF, we intended to use the clinical data of patients with IPF before the era of antifibrotic therapy to exclude the antifibrotic effect. This enabled comparison of our data with the previous results. Despite these limitations, our study could demonstrate clinical validity of the revised definition of AE in patients with IPF.

In conclusion, per the revised definition, approximately a fifth of patients with IPF experienced AE which exerted a significant impact on the prognosis. Our results suggest that the revised definition reflects 20.4% increase of incidence and 10% reduction of the in-hospital mortality in AE defined by the past definition, by reducing suspected cases of AE and including triggered cases. These results must be considered when interpreting past results and planning future clinical trials.

## Supplementary Information

Supplementary Information.
