# Supplementary material for: Impact of the revised definition on incidence and outcomes of acute exacerbation of idiopathic pulmonary fibrosis
Source: Sci Rep. 2022 May 25;12:8817. doi: 10.1038/s41598-022-12693-5 (PMC9130993; doi:10.1038/s41598-022-12693-5)
Supplement: Supplementary file 1 — Supplementary Information. [file 41598_2022_12693_MOESM1_ESM.docx]

**SUPPLEMENTRY INFORMATION**

**Impact of the revised definition on incidence and outcomes of acute exacerbation in idiopathic pulmonary fibrosis**

Jung-Wan Yoo, MD^1#^, Jehun Kim, MD^2#^, Jin Woo Song, MD, PhD^2^

^1^Department of Internal Medicine, Gyeongsang National University Hospital, Jinju, Republic of Korea

^2^Department of Pulmonary and Critical Care Medicine, Asan Medical Center, University of Ulsan College of Medicine, Seoul, Republic of Korea.

**Figure S1** Flow chart depicting enrollment of patients.

**
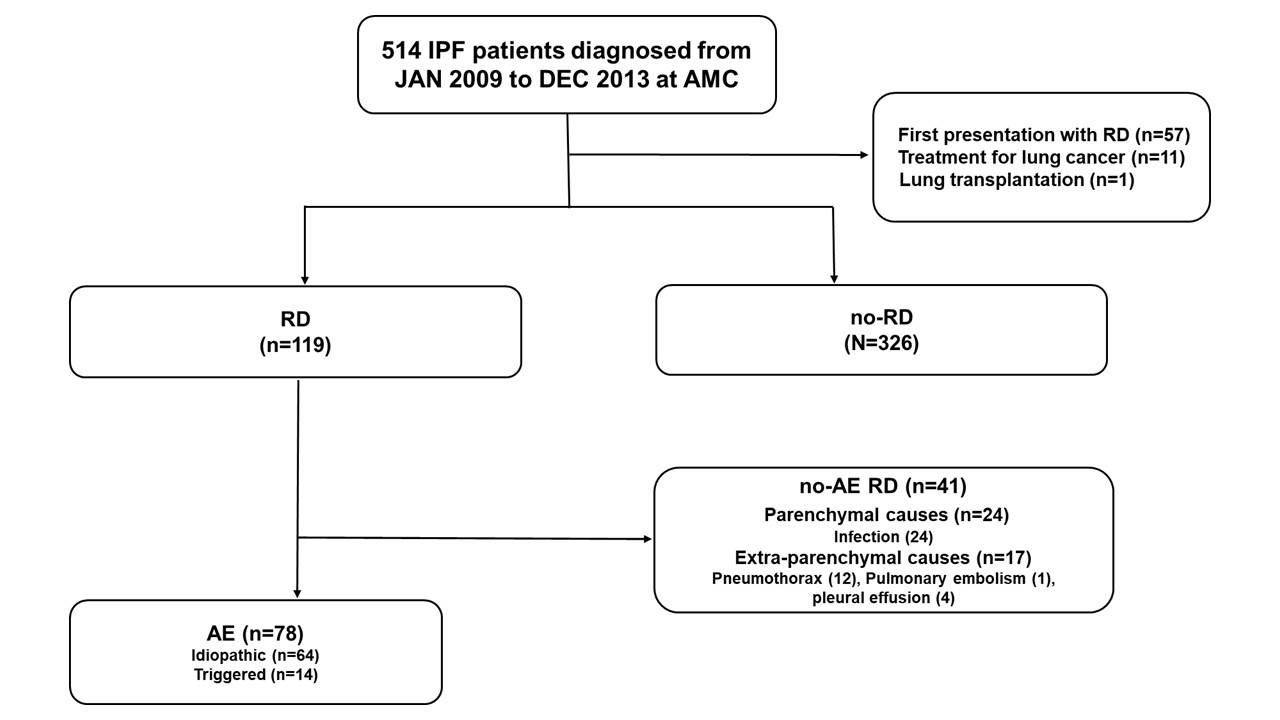
**

IPF: idiopathic pulmonary fibrosis, AMC: Asan Medical Center, RD: acute respiratory deterioration, AE: acute exacerbation

**Figure S2** Frequency of RD and AE in patients with IPF.

**
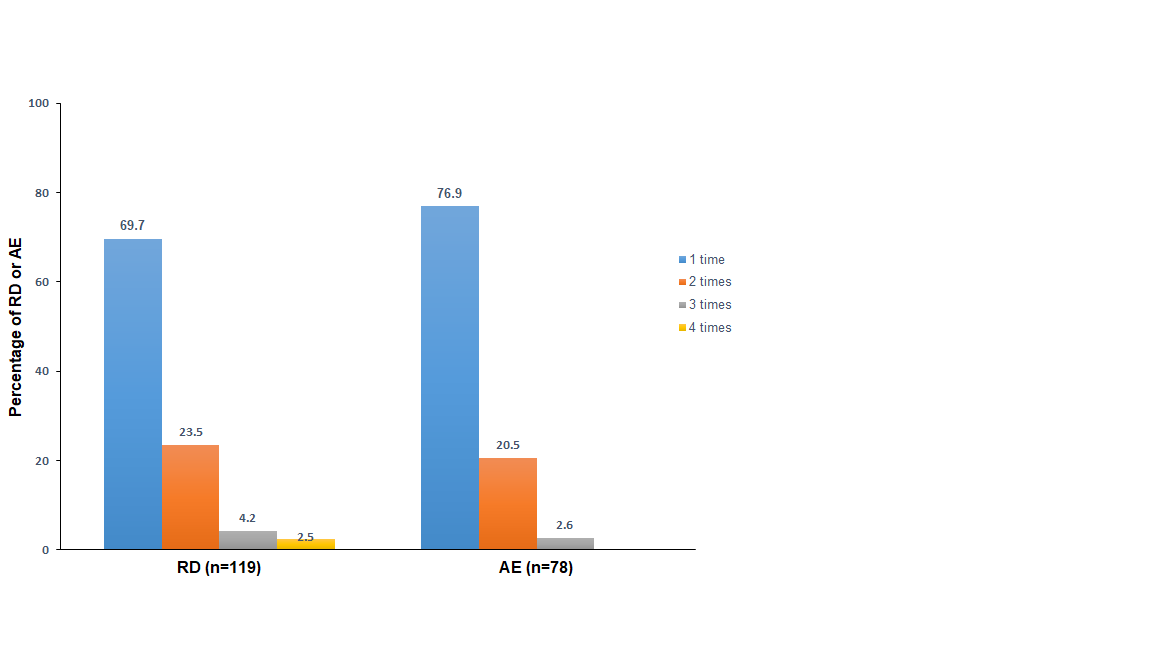
**

The number on the right side indicates frequency of RD or AE. RD, acute respiratory deterioration; AE, acute exacerbation; IPF, idiopathic pulmonary fibrosis

**Figure S3** The seasonal frequency of AE in patients with IPF.

*
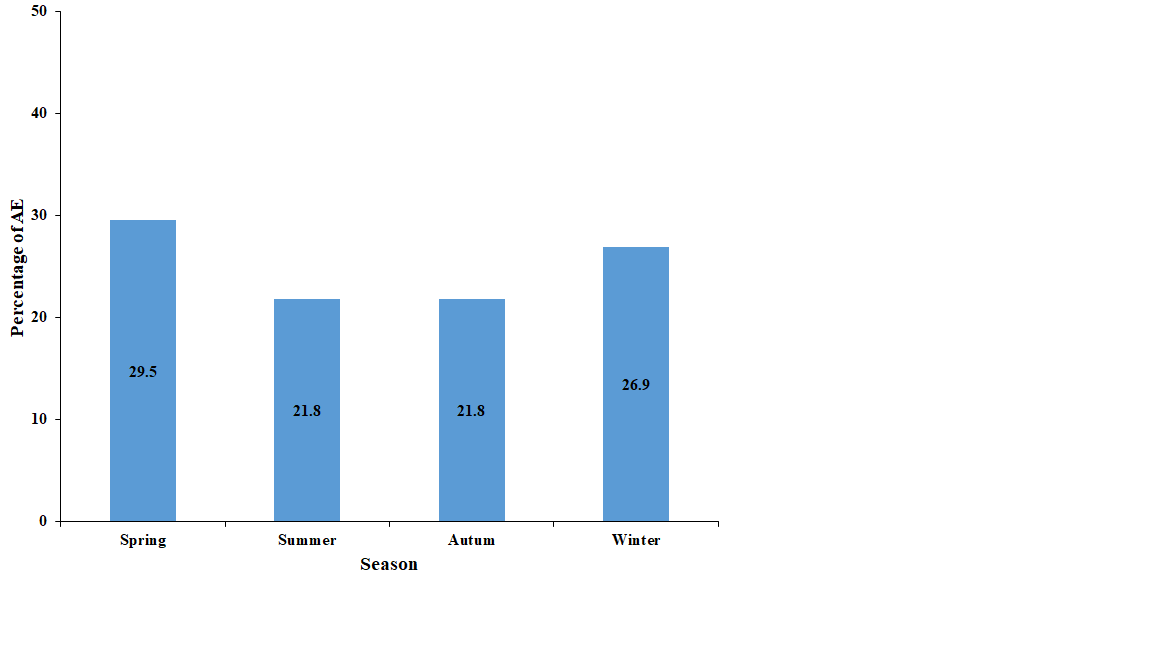
*

AE, acute exacerbation; IPF, idiopathic pulmonary fibrosis

**Figure S4** Comparison of survival curves from hospitalization between idiopathic and triggered AE groups among IPF patients with AE.

*
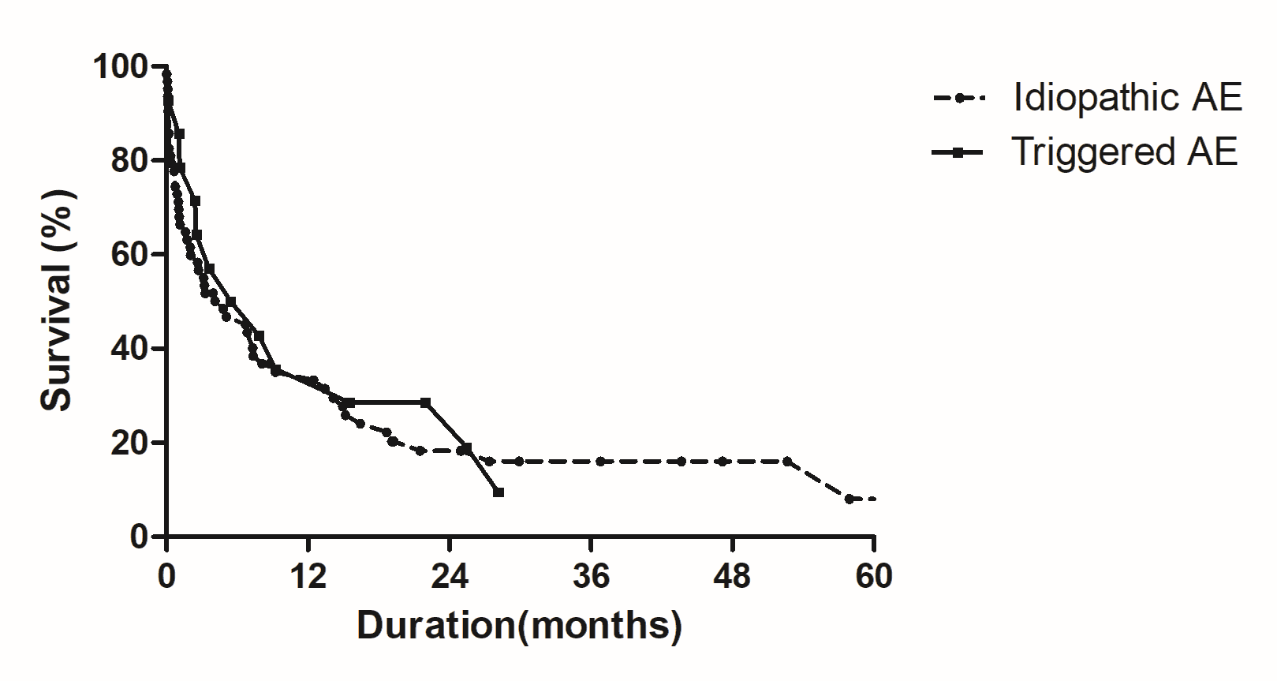
*

AE: acute exacerbation, IPF: idiopathic pulmonary fibrosis

**Table S1** Etiologies of RD in patients with IPF.

| Etiology | Case, N (%) | Documented organisms |
| --- | --- | --- |
| Total RD | 119 |  |
| Bilateral lesions | 79 (66.4) |  |
| AE | 78 (65.5) |  |
| Idiopathic | 64 (53.8) |  |
| Triggered | 14 (11.8) |  |
| Infection | 12 (10.1) | Bacteria* (4), virus† (6),  mycobacteria‡ (1), fungus^#^ (1) |
| Post-operation | 1 (0.8) |  |
| Drug toxicity | 1 (0.8) |  |
| Pulmonary embolism | 1 (0.8) |  |
| Unilateral lesion | 40 (33.6) |  |
| Infection | 24 (20.2) | Mycobacteria‡ (1), fungus^#^ (2) |
| Pneumothorax | 12 (10.1) |  |
| Pleural effusion | 4 (3.4) |  |

Data are expressed as a number (percent). RD, acute respiratory deterioration; IPF, idiopathic pulmonary fibrosis; AE, acute exacerbation. Infection was identified in 4 sputum , 5 BAL fluid, 1 pneumococcal urinary Antigen test, 2 positive IgM serology for mycoplasma pneumonia).

*Bacteria*: Streptococcus pneumonia* (1), *Mycoplasma pneumonia* (2), *Moraxella catarrhalis* (1), †Virus: Rhinovirus (3), respiratory syncytial virus (1), Metapneumovirus (1), adenovirus (1), ‡Mycobacteria: *Mycobacterium intracellulare* (2), ^#^Fungus: Aspergillus species (3)

**Table S2** Comparison of clinical features at the time of hospitalization between the survivors and the non-survivors among IPF patients with AE.

| Variables | Survivors | Non-survivors | *P* value |
| --- | --- | --- | --- |
| No. of patients | 55 | 23 |  |
| Age, years | 71.8±7.3 | 71±8.7 | .694 |
| Male gender | 42 (76.4) | 14 (60.9) | .267 |
| Ever smoker | 36 (65.5) | 15 (65.2) | .315 |
| Duration of dyspnea, days | 13.9 ± 13.5 | 7.0 ± 7.7 | .006 |
| Cough | 45 (81.8) | 20 (87) | .824 |
| Sputum | 39 (70.9) | 16 (69.6) | >.999 |
| Fever | 13 (23.6) | 12 (52.2) | .028 |
| CRP, mg/dl | 6.7 ± 7.6 | 12.8 ± 8.2 | <.001 |
| P/F ratio | 290.9 ± 117.9 (n=53) | 150.8 ± 78.7  (n=23) | <.001 |
| Steroid ± IM (before AE)^a^ | 19 (34.5) | 13 (56.5) | .072 |

Data are expressed as a mean ± standard deviation or a number (%) unless otherwise indicated.

IPF, idiopathic pulmonary fibrosis; AE, acute exacerbation; CRP, C-reactive protein; P/F, partial pressure of arterial oxygen/fraction of inspired oxygen; IM, immunosuppressant.

^a^: Azathioprine (n=11), Mycophenolate mofetil (n=2), Cyclosporine (n=5).

**Table S3** Comparison of treatment between the survivors and non-survivors during hospitalization among IPF patients with AE

| Treatment | Total | Survivors | Non-survivors | *P*-value |
| --- | --- | --- | --- | --- |
| No. of patients | 78 | 55 | 23 | 0.389 |
| Steroid pluse^a^ | 4 (5.1) | 3 (5.5) | 1 (4.3) |  |
| Steroid pulse plus IM^b^ | 5 (6.4) | 4 (7.3) | 1 (4.3) |  |
| High-dose steroid^c^ | 31 (39.7) | 17 (30.9) | 14 (60.9) |  |
| High-dose steroid plus IM | 7 (9) | 6 (10.9) | 1 (4.3) |  |
| Low dose steroid^d^ | 6 (7.7) | 5 (9.1) | 1 (4.3) |  |
| Low-dose steroid plus IM | 4 (5.1) | 3 (5.5) | 1 (4.3) |  |
| No steroid and IM | 21 (26.9) | 17 (30.9) | 4 (17.4) |  |

Data are expressed as numbers (percentages). AE, acute exacerbation; IPF, idiopathic pulmonary fibrosis ; IM, immunosuppressive agent.

^a^Steriod pulse was defined as 500 mg/day or more of methylprednisolone for 3 – 5 days.

^b^IM was azathioprine, mycophenolate mofetil, or cyclosporine.

^c^High-dose steroid was defined as 0.5 mg/kg/day or more of methylprednisolone.

^d^Low-dose steroid was defined as less than 0.5mg/kg/day of methylprednisolone.

**Table S4** Prognostic factors for in-hospital mortality in IPF patients with AE assessed by using logistic regression analysis

| Variables | Unadjusted | | | | Multivariable | | | | |
| --- | --- | --- | --- | --- | --- | --- | --- | --- | --- |
|  | | OR | 95% CI | *P* value | | OR | 95% CI | *P* value |  |
| Age | | 0.987 | 0.926-1.052 | .690 | |  |  |  |  |
| Male gender | | 0.481 | 0.170-1.367 | .170 | |  |  |  |  |
| Dyspnea duration | | 0.933 | 0.875-0.994 | .033 | | - | - | - |  |
| Ever smoker | | 0.990 | 0.356-2.751 | .984 | |  |  |  |  |
| Cough | | 1.481 | 0.368-5.969 | .580 | |  |  |  |  |
| Sputum | | 0.938 | 0.324-2.712 | .906 | |  |  |  |  |
| Fever | | 3.524 | 1.261-9.850 | .016 | | 3.342 | 0.959-11.648 | .058 |  |
| CRP | | 1.092 | 1.027-1.162 | .005 | | - | - | - |  |
| P/F ratio | | 0.987 | 0.980-0.993 | < .001 | | 0.987 | 0.980-0.994 | <.001 |  |
| Steroid ± IM (before AE) | | 2.463 | 0.911-6.657 | .076 | | - | - | - |  |

IPF, idiopathic pulmonary fibrosis; AE, acute exacerbation; OR, odds ratio; CI, confidence interval; CRP. C-reactive protein; P/F, partial pressure of arterial oxygen/fraction of inspired oxygen; IM, immunosuppressant.

**Table S5** Prognostic factors at the time of hospitalization for in-hospital mortality of IPF patients with AE defined by the past criteria^a^

| Variables | Unadjusted | | | | Multivariable | | |
| --- | --- | --- | --- | --- | --- | --- | --- |
|  | OR | 95% CI | *P* value | OR | | 95% CI | *P* value |
| Age | 0.968 | 0.905-1.036 | 0.343 |  | |  |  |
| Male | 0.371 | 0.115-1.195 | 0.097 | - | | - | - |
| Dyspnea duration | 0.934 | 0.872-0.999 | 0.048 | - | | - | - |
| Ever smoker | 0.867 | 0.284-2.647 | 0.802 |  | |  |  |
| Cough | 4.571 | 0.532-39.254 | 0.166 |  | |  |  |
| Sputum | 1.387 | 0.419-4.588 | 0.592 |  | |  |  |
| Fever | 3.630 | 1.195-11.024 | 0.023 | 3.698 | | 0.927-12.182 | 0.063 |
| CRP | 1.127 | 1.041-1.219 | 0.003 | - | | - | - |
| P/F ratio | 0.986 | 0.979-0.994 | <0.001 | 0.987 | | 0.979-0.994 | 0.001 |
| Steroid ± IM (before AE) | 2.053 | 0.710-5.935 | 0.184 |  | |  |  |

IPF, idiopathic pulmonary fibrosis; AE, acute exacerbation; OR, odd ratio; CI, confidence interval; CRP, C-reactive protein; P/F, partial pressure of arterial oxygen/fraction of inspired oxygen; CRP, C-reactive protein; IM, immunosuppressant

^a^ AE was defined by the 2007 criteria.

**Table S6** Prognostic factors for overall mortality in patients with IPF assessed by using Cox regression analysis

| Variables | Unadjusted | | | | Multivariate | | | |
| --- | --- | --- | --- | --- | --- | --- | --- | --- |
|  | | HR | 95% CI | *P* value | | HR | 95% CI | *P* value |
| Age | | 1.040 | 1.02-1.060 | <0.001 | | 1.029 | 1.006-1.052 | 0.013 |
| Male | | 1.016 | 0.735-1.403 | 0.924 | |  |  |  |
| BMI | | 0.908 | 0.865-0.953 | <0.001 | | 0.935 | 0.886-0.987 | 0.014 |
| Charlson comorbidity index | | 1.130 | 1.018-1.255 | 0.022 | |  |  |  |
| Ever smoker | | 1.045 | 0.771-1.416 | 0.777 | |  |  |  |
| FVC, % pred | | 0.946 | 0.937-0.956 | <0.001 | | 0.970 | 0.957-0.983 | <0.001 |
| DLco, % pred | | 0.951 | 0.943-0.960 | <0.001 | | 0.987 | 0.973-1.001 | 0.066 |
| 6MWT distance | | 0.994 | 0.993-0.996 | <0.001 | | 0.998 | 0.996-1.000 | 0.019 |
| 6MWT resting SpO_2_ | | 0.791 | 0.728-0.858 | <0.001 | | - | - | - |
| 6MWT lowest SpO_2_ | | 0.968 | 0.960-0.976 | <0.001 | | 0.972 | 0.956-0.988 | 0.001 |
| Disease progression^a^ | | 3.043 | 2.169-4.267 | <0.001 | | 2.412 | 1.687-3.449 | <0.001 |
| AE^b^ | | 3.254 | 2.339-4.527 | <0.001 | | 2.978 | 1.433-6.190 | 0.003 |
| Steroid ± IM | | 1.057 | 0.767-1.457 | 0.734 | | - | - | - |

IPF, idiopathic pulmonary fibrosis; HR, hazard ratio; CI, confidence interval; BMI, body mass index; FVC, forced vital capacity; DLco, diffuse lung capacity of carbon monoxide; 6MWT, 6-minute walk test; SpO_2_, saturation of pulse oximetry; AE, acute exacerbation; IM, immunosuppressant.

^a^ Disease progression defines 10% relative decline in FVC for 6 months.

^b^ AE was defined by the 2007 criteria.
